# Negative Magnetic Sorting Preserves the Functionality of Ex Vivo Cultivated Non-Adherent Human Monocytes

**DOI:** 10.3390/biology11111583

**Published:** 2022-10-27

**Authors:** Melanie Hornschuh, Vivian Haas, Paul P. Winkel, Mira Y. Gökyildirim, Christina S. Mullins, Ida Maria Wrobel, Christian Manteuffel, Elisa Wirthgen

**Affiliations:** 1 Department of Pediatrics, Rostock University Medical Center, 18057 Rostock, Germany; 2 Medical School, Rostock University, 18057 Rostock, Germany; 3 Department of General Surgery, Patient Models for Precision Medicine, Rostock University Medical Center, 18057 Rostock, Germany; 4 Department of Transfusion Medicine, Rostock University Medical Center, 18057 Rostock, Germany; 5 Institute of Behavioral Physiology, Research Institute for Farm Animal Biology, 18196 Dummerstorf, Germany

**Keywords:** monocytes, positive magnetic sorting, negative magnetic sorting, cellular therapy

## Abstract

**Simple Summary:**

Immune cells are of increasing interest for cellular therapeutic products to treat inflammation-related diseases and cancer. However, the isolation method and the culture conditions influence the cells’ functionality. In contrast to dendritic cells and macrophages, the effects of the isolation method on the functionality of non-adherent peripheral monocytes have not yet been investigated. Hence, the present study examines the impact of the isolation method on cell viability, growth, metabolism, inflammation-induced cytokine response, migratory capacity, and adherence of non-adherent human peripheral monocytes. Monocytes were isolated by magnetic sorting using either positive or negative selection and cultured plates, which prevented the adhesion of the monocytes. The purity and yield of monocytes were higher after positive selection, confirming previous findings. However, the adherence and migratory capacity, cytokine response, and metabolic activity were decreased compared to negatively selected monocytes. The impaired functionality, combined with cell shrinking, thus, indicates the start of cell viability loss. Negatively selected non-adherent monocytes showed no impairment in functionality, and the viability remained high. In conclusion, this approach is better suited for conducting ex vivo modifications of monocytes prior to the intended experimental setup or therapeutic application.

**Abstract:**

Background: Monocyte-derived macrophages or dendritic cells are of increasing interest for cellular therapeutic products to treat inflammation-related diseases and cancer. However, the isolation method and the culture conditions applied influence the functionality of cells. For some approaches, the adhesion-induced differentiation into macrophages must be prevented to maintain functions attributed to circulating monocytes. The effects of the isolation method on the functionality of non-adherent peripheral monocytes have not yet been investigated. Methods: The present study examines the impact of the isolation method on cell viability, growth, metabolism, inflammation-induced cytokine response, migratory capacity, and adherence of non-adherent human peripheral monocytes. The monocytes were isolated by magnetic sorting using either positive or negative selection and cultured in cell-repellent plates. Results: The purity and yield of monocytes were higher after positive selection. However, the adherence and migratory capacity, cytokine response, and metabolic activity were decreased compared to negatively selected monocytes. The impaired functionality presented in combination with cell shrinking, thus, indicates the start of cell viability loss. Negatively selected non-adherent monocytes showed no impairment in functionality, and the viability remained high. In conclusion, this approach is better suited for conducting ex vivo modifications of monocytes prior to the intended experimental setup or therapeutic application.

## 1. Introduction

Monocytes from the peripheral blood play a crucial role in producing inflammatory mediators and regulating the innate and adaptive immune response [1,2]. They are recruited from the peripheral blood to mucosal tissues or sites of inflammation, where they subsequently differentiate into macrophages or dendritic cells, releasing inflammatory mediators [1,2]. The migration of circulating monocytes into surrounding tissues occurs more frequently during inflammation, as monocytes are specifically attracted by proinflammatory mediators such as chemokines, complement components, and released tissue components. Monocytes and macrophages also play an important role in maintaining intestinal homeostasis by clearing bacterial antigens and apoptotic cells and enhancing or inhibiting the action of other immune cells, depending on the prevailing microenvironment. Due to their migratory capacity and specific mode of action, monocytes are of increasing interest for use as cellular therapeutics for inflammation-related diseases and cancer [3,4,5,6,7,8]. The possibility of using ex vivo modified monocytes to treat arteriogenesis was already investigated in 2006 by Herold et al. [3]; however, this group also pointed out problems with isolation and the culture of these cells. The assumed therapeutic mode of action is that the genetically or biochemically modified monocytes are recruited to the site of inflammation after injection, followed by migration into inflamed tissue and exertion of their intended effect. An ex vivo expansion of isolated blood monocytes, as currently described for T cells [9,10], is not feasible according to the current state of the art since circulating monocytes do not proliferate in the absence of interaction with other cell types [11,12]. Therefore, it is crucial to keep the yield of cultured monocytes as high as possible and to maintain vitality and functionality. In this context, the isolation procedure may have detrimental effects on the intended use as a cellular product. Besides monocyte isolation by adherence [13] or density centrifugation [14,15,16,17], magnetic sorting by positive or negative selection is a common way to isolate cells from multi-cell-suspension, i.e., buffy coats (BC). During positive selection, all monocytes are isolated with magnetic beads coupled with antibodies against the CD14 surface molecules of monocytes. The bead-coupled antibodies are added to the cell suspension, which then flows through a magnetic column that retains the labeled cells. An advantage of this isolation is the high purity due to the selective binding of the antibodies [18]. Nevertheless, in ex vivo generated monocyte-derived dendritic cells, functions such as phagocytosis and cytokine response were altered by this approach. Moreover, studies in a human leukemia monocytic cell line revealed an impaired secretion of proinflammatory cytokines in response to lipopolysaccharides (LPS) [19]. This is presumed to be an effect of the antibody-engagement during isolation [19,20,21]. The process of negative selection is characterized by magnetic labeling of all cells except the monocytes; thus, the monocytes remain “untouched”. However, any falsely unlabeled cell will affect the purity by “contaminating” the cell suspension. 

Following the above-mentioned aspects, this study investigated the effect of the isolation method on purity and viability with a particular interest in monocyte-specific functions. These parameters may be associated with the functionality of monocytes intended for application in cell-based therapeutic products. In contrast to other methodological studies [18,20], we prevented the adherence of monocytes by using cell-repellent surfaces as previously described [22] to avoid adhesion-induced differentiation into macrophages [23]. In particular, our study aimed to preserve the migratory capacity of the monocytes, which is severely limited with longer culture duration [22] and under adherent culture conditions [24]. Furthermore, we evaluated the effects of the isolation method on unstimulated (naïve) and activated monocytes. The activation of monocytes was induced experimentally by the granulocyte-macrophage colony-stimulating factor (GM-CSF), which is often applied to the culture of mature myeloid cells as it acts as a pro-survival and activating factor [25,26]. According to previous results, the activation was confirmed by the measurement of interleukin-8 (IL-8) and tumor necrosis factor-alpha (TNF-α) [22]. Moreover, we evaluated the effect of isolation method on LPS responsiveness by measuring the TNF-α secretion.

## 2. Materials and Methods

### 2.1. Human Monocytes

Monocytes were isolated from a total of 19 buffy coat (BC) samples, provided by the Department of Transfusion Medicine, Rostock University Medical Center, Rostock, after written consent for scientific use (approval number: A2011-140, Rostock University Ethics committee) and prepared as previously described [22]. At the time of monocyte isolation, the BCs were no more than 4 h old.

### 2.2. Experimental Design

**Figure 1:**
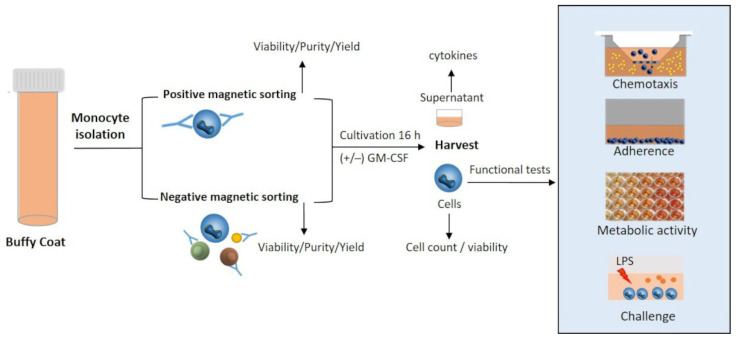
Experimental design. Monocytes were isolated from human BCs either via positive or negative magnetic sorting. Subsequently, the monocytes were cultivated ex vivo with or without GM-CSF. After 16 h, cells and supernatants were harvested for assessment. *Abbr.: GM-CSF, granulocyte-macrophage colony-stimulating factor; LPS, lipopolysaccharides*.

An overview of the applied experimental design is illustrated in Figure 1. Monocytes were isolated from human BCs either by positive or negative selection. The purity of CD14^+^ cells was analyzed by flow cytometry directly after isolation, as previously described [22]. After isolation, the cells were adjusted to a final concentration of 1 × 10^6^ viable cells/ml and seeded in 24-well cell-repellent plates (Greiner Bio-One, Leipzig, Germany). Both activated and naïve monocytes were investigated. The activation of monocytes was experimentally induced by GM-CSF (10 ng/ml, PAN-Biotech, Aidenbach, Germany), as described previously [6,22], while naïve cells remained unstimulated. The monocytes were cultivated overnight for 16 h at 37 °C (95% humidity). Longer cultivation duration was associated with a loss of migratory capacity [22] and, therefore, was not investigated in this study. After cultivation, the monocytes were harvested and examined in various tests to analyze their functionality (adherence, metabolic activity, LPS response, migratory capacity). Furthermore, the supernatants were collected and stored at −20 °C until cytokine measurement. After isolation and culturing of monocytes, the cells’ number, size, and viability were evaluated using the CASY cell counter according to the manufacturer’s instructions [22].

### 2.3. Monocyte Isolation by Positive and Negative Selection

Monocyte isolation via positive selection was performed according to the manufacturer’s protocol StraightFrom^®^ Whole Blood CD14 MicroBeads (human, Miltenyi Biotec, Bergisch Gladbach, Germany). The negative selection was performed using the EasySep^TM^ Direct Human Monocyte Isolation Kit (STEMCELL, Vancouver, BC, Canada) as previously described [22]. After the isolation, monocytes were diluted in RPMI 1640 medium (specification: very low endotoxin), supplemented with 2 mM stable glutamine, 10% fetal bovine serum, 100 U/mL penicillin, and 0.1 mg/mL streptomycin (all: PAN-Biotech, Aidenbach, Germany). The cells were seeded in cell-repellent multiwell plates with a concentration of 1 × 10^6^ cells/ml and incubated at 37 °C and 5% CO_2_ according to the applied experimental design.

### 2.4. Flow Cytometry Analysis

For flow cytometry analyses, the cells were stained with anti-human CD14 antibodies (PerCP-Vio700-A, Biolegend, San Diego, CA, USA). The live-dead staining was performed with the Zombie NIR™ Fixable Viability Kit according to the manufacturer’s instructions to determine the viability of the CD14^+^ subset (BioLegend, San Diego, CA, USA). After washing, Fc receptor blocker (Human True stain FcX^TM^, Biolgend) and monocyte blocker (True-Stain Monocyte Blocker^TM^, Biolegend) were added to the cells and incubated for 10 min (dark, room temperature) to prevent unspecific antibody binding. Subsequently, the cells were incubated with CD14 antibodies for 20 min at 4 °C, followed by two wash steps. The cell pellet was dissolved in FACS buffer for measurement at the Cytek™ Aurora flow cytometer (Cytek Biosciences, Amsterdam, The Netherlands). The analysis of the data was performed with *FlowJo* software, Version 10.8.1 (BD Life Sciences, Ashland, MA, USA).

### 2.5. Migratory Capacity, Adherence, Metabolic Activity

After culturing for 16 h, naïve and activated monocytes were harvested to evaluate their functionality as described in detail by Wirthgen et al. [22]. In brief, a chemotaxis assay was performed immediately after cultivation. Monocytes were harvested and seeded in duplicates into 5 µm TC-Inserts (500,000 viable cells/insert) (Sarstedt, Nümbrecht, Germany). The inserts were placed in duplicates in a 24-well culture plate (Greiner-Bio-One, Leipzig, Germany), prepared with the cell culture medium with or without the chemoattractant MCP-1 (100 nml, R&D systems, Minneapolis, MN, USA). The number of cells in the lower chamber was measured to calculate the number of cells that had migrated from the TC-Insert to the lower chamber. In the adherence assay, the monocytes were seeded in triplicates in fibronectin-coated 96-well culture plates (100,000 viable cells/well) and incubated for 2.5 h. Adherent cells were detached by incubation with accutase (PAN-Biotech, Aidenbach, Germany) at room temperature for 20 min. Monocytes were then counted using the CASY cell counter. To measure the metabolic activity of the cells, monocytes were transferred to a 96-well culture plate (triplicates). WST-1 reagent ([2-(4-Iodophenyl)-3-(4-nitrophenyl)-5-(2,4-disulfophenyl)-2H-tetrazolium], Merck, Darmstadt, Germany) was added according to the manufacturer’s instructions. After incubation for 60 min, the optical density was measured at 440/650 nm using the TECAN micro-plate reader (Infinite m200, TECAN, Männedorf, Schweiz). 

### 2.6. Response to Lipopolysaccharides

Naïve and activated monocytes were harvested after 16 h of cultivation and seeded into a 96-well plate (100,000 viable cells/well, in triplicate). The cells were stimulated with LPS (E. Coli O111:B4, Sigma-Aldrich, Taufkirchen, Germany) for 24 h at a final concentration of 50 ng/mL. The proinflammatory cytokine TNF-α secretion was measured in the cell culture supernatants since it was shown to be highly responsive to LPS [27,28]. 

### 2.7. Quantification of Cytokines

Concentrations of TNF-α and IL-8 were analyzed in cell culture supernatants using commercially available human enzyme-linked immunosorbent assays (ELISAs) (DuoSet^®^ ELISA Kits, R&D systems, Minneapolis, MN, USA). Analyses were performed according to the manufacturer’s instructions.

### 2.8. Statistical Analyses

Statistical analyses were performed using GraphPad Prism version 9.2.0 (GraphPad Software Inc., San Diego, CA, USA). Fixed effects on the continuous response variables IL-8, TNF-α, and metabolic activity were analyzed by Analysis of Variance (ANOVA, results not presented). Univariate analyses and pairwise comparisons of two groups were performed with Student’s t-test (parametric distribution) or Wilcoxon rank test (non-parametric distribution). GraphPad Prism provides no means to perform an ANOVA or comparisons for multivariate models with response variables from the closed [0, 1] interval (cell size, adherence, and migratory capacity). Therefore, such analyses were performed using generalized linear mixed models assuming a binomial distribution and using the logit link function. The analysis was performed with “R” statistics software Version 3.6.2 [29] using the emmeans package [30] for multiple comparisons of least-square mean estimates. In any case, multiple pairwise comparisons were corrected using the Tukey–Kramer procedure [31]. Asterisks mark significant differences, whereby *p* < 0.05, *p* < 0.01, and *p* < 0.001 were chosen as levels of significance. Differences that are not informative for the research question are not presented in the text and figures (e.g., positive isolated cells + GM-CSF vs. negative isolated cells w/o GM-CSF).

## 3. Results

### 3.1. Yield, Viability, and Purity Directly after Monocyte Isolation

**Figure 2:**
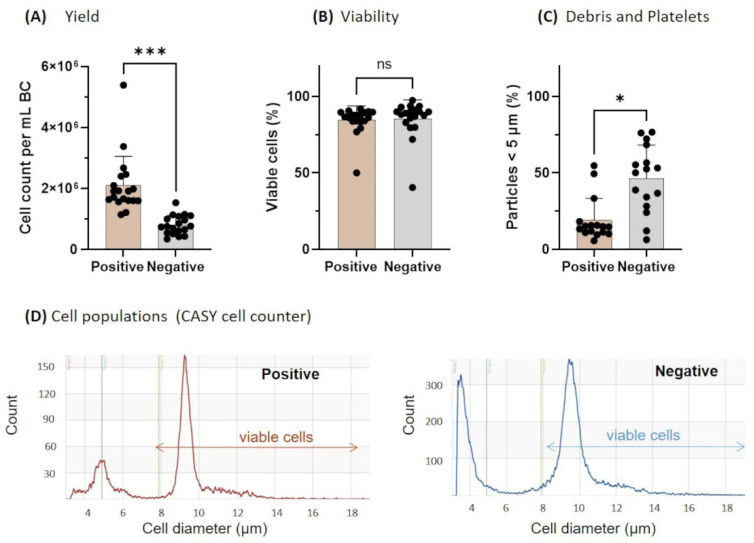
Effect of positive and negative isolation on yield (**A**), viability (**B**), and debris/platelets (**C**) measured by CASY cell counter and calculated as described [22]. A representative cell count vs. cell size profile after positive or negative selection is shown in (**D**). (**A**–**C**): Results are presented as single dots for each BC and as Means + SD. Pairwise comparisons were calculated with the Wilcoxon matched-pairs signed rank test. (**A**–**D**): n = 19; *** *p* < 0.001, * *p* < 0.05, ns = not significant.

**Figure 3:**
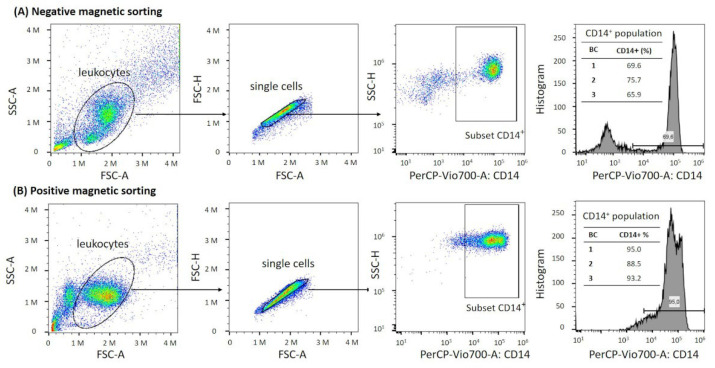
Gating strategy for quantifying the percentage of CD14^+^ monocytes from the leukocyte population after negative magnetic sorting (**A**) and positive magnetic sorting (**B**). The viable leukocyte population was first individualized by crossing the singlet gate and gating on the CD14^+^ gate. The threshold for CD14^+^ cells was evaluated by data from the histograms and unstained controls. The respective histogram of BC1 is shown as an example. The tables show the percentage of CD14^+^ monocytes of viable leukocytes for each BC. *Abbr.: SSC-A, side scatter area; FSC-A, forward scatter area, CD14, cluster of differentiation 14*.

The total yield of cells perml of buffy coat measured with the CASY cell counter was significantly higher in the positively selected cases (Figure 2A), while the viability was comparable for both positive and negative selection (Figure 2B). The count of particles with a diameter < 5 µm including cell debris and platelets was higher in negatively isolated cell suspensions (Figure 2C). The peak width of viable cells, created by the CASY Software, was more narrow after positive selection, indicating a higher purity of the monocyte population compared to negatively selected cells (exemplary plots in Figure 2D). Since CASY is not able to detect cell-specific surface markers for monocytes, additional flow cytometry analyses of three isolated cell suspensions were performed by examining CD14 as a classical monocyte marker. Flow cytometry analyses confirmed a higher proportion of CD14^+^ monocytes within the viable leukocyte population after the positive selection (mean: 92.2%) compared to negatively selected monocytes (mean: 70.3%). Representative plots and the gating strategy used to evaluate the proportion of CD14^+^ monocytes are presented in Figure 3A,B.

### 3.2. Functionality of Naïve and Activated Monocytes after Cultivation

**Figure 4:**
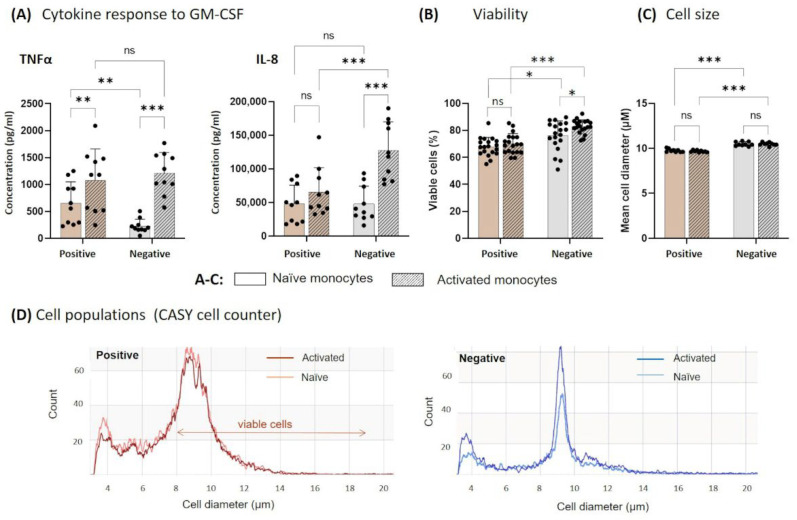
Effect of isolation method on cytokine response, viability, cell size, and functionality of blood-derived monocytes. After isolation from whole blood, monocytes were cultured ex vivo with GM-CSF (=activated) or left unstimulated (=naïve) for 16 h in suspension culture. (**A**) The non-adherent cells were collected, and secreted cytokines TNF-α and IL-8 were measured in cell culture supernatants by ELISA. (**B**) The viability and the cell size (**C**) were measured using the CASY cell counter. (**D**) A representative result of the gated monocyte population is shown based on cell diameter and cell counts. Results are presented as single dots representing one sample. Bars indicate means + SD. Pairwise comparisons were calculated using the Tukey–Kramer test. (**A**): n = 10, (**B**,**C**): n = 19; *** *p* < 0.001, ** *p* < 0.01, * *p* < 0.05., ns = not significant *Abbr.: GM-CSF, granulocyte-macrophage colony-stimulating factor; TNF-α, tumor necrosis factor alpha; IL-8, interleukin 8*.

The isolation method and the GM-CSF treatment affected the secretion of TNF-α and IL-8 of the non-adherent monocytes. TNF-α levels were significantly increased in supernatants of positively isolated naïve monocytes compared to the supernatants of negatively isolated monocytes. The activation with GM-CSF significantly increased the secretion of TNF-α up to a similar level in both isolation methods (Figure 4A). The isolation method did not significantly affect the IL-8 secretion of naïve monocytes. A GM-CSF-dependent response of IL-8 secretion was seen in the supernatant of positively and negatively selected monocytes. However, the concentration of IL-8 was significantly increased in activated monocytes after negative isolation (Figure 4A). The percentage of viable cells was generally higher after negative isolation. The activation with GM-CSF increased the number of living cells after the 16 h cultivation duration (Figure 4B). The mean cell diameter of viable cells was lower in cells isolated by positive selection (Figure 4C). 

A shrinkage of cell volume after positive isolation was also visible in the CASY profile of cell size distribution, exemplarily shown in Figure 4D. After positive selection, a shift of monocytes towards the range of dead cells (<7.9 µm) and cell debris (<5 µm) appeared. This was not detected in negatively selected monocytes. 

**Figure 5:**
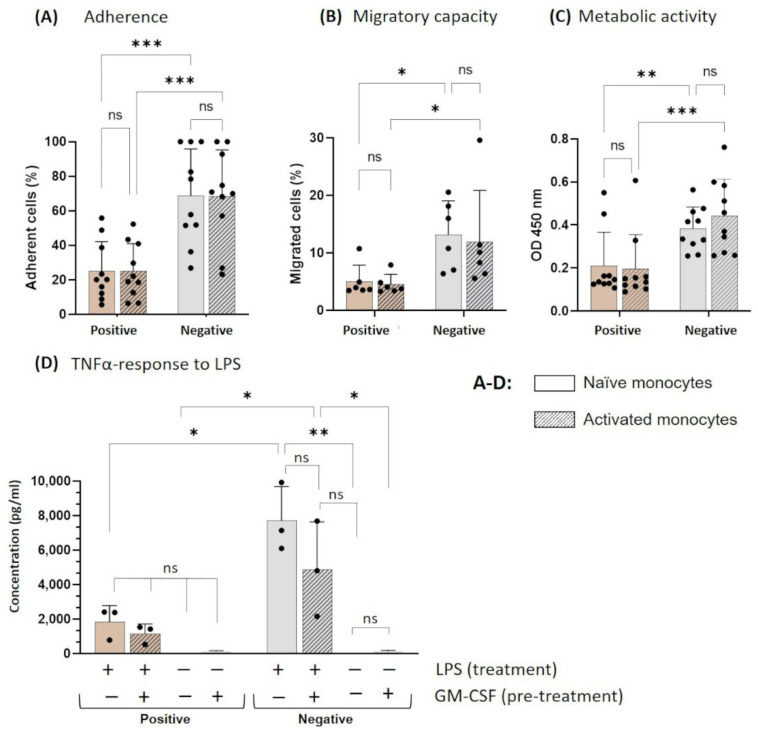
Effect of isolation method on the functionality of non-adherent blood-derived monocytes. After isolation from whole blood, monocytes were cultured for 16 h in suspension culture with GM-CSF (=activated) or left unstimulated (=naïve), followed by the harvest of the non-adherent cells. (**A**) Monocytes were allowed to adhere to fibronectin-coated multiwell plates for 2.5 h to measure the effects on cell adhesion. Adherent cells were enzymatically detached and counted. (**B**) Monocytes were seeded in a transwell insert to determine the migratory capacity towards the MCP-1 supplemented medium. After a 2.5 h incubation period, the cells in the lower compartment of the chamber were counted, and the ratio to the number of seeded cells was calculated and indicated as a percentage. (**C**) The metabolic activity of cells was examined in multiwell plates using WST-1 assay. (**D**) The secreted cytokine TNF-α was measured in cell culture supernatants after 24 h of LPS or PBS stimulation by ELISA. Results are presented as single dots representing one sample. Bars indicate means + SD. Pairwise comparisons were calculated using the Tukey–Kramer test. (**A**): n = 10, (**B**): n = 6, (**C**): n = 10, (**D**): n = 3, * *p* < 0.05, ** *p* < 0.01, ***, *p* < 0.001, ns = not significant *Abbr.: GM-CSF, granulocyte-macrophage colony-stimulating factor; LPS, lipopolysaccharides; TNF-α, tumor necrosis factor-alpha*.

After cultivation, the monocytes were harvested and tested for their capacity to adhere and migrate. Additionally, their response to bacterial-derived stimuli was assessed. Irrespective of GM-CSF pre-treatment, the adherence of negatively selected monocytes was roughly three times higher than in positively selected monocytes (Figure 5A). The lower adherence in positively isolated monocytes was associated with a reduced migratory capacity (Figure 5B) and metabolic activity (Figure 5C) compared to negatively isolated cells. In our study, GM-CSF activation did not affect adherence, migratory capacity, or metabolic activity of isolated monocytes. In an additional assay, the harvested naïve and activated monocytes were challenged with LPS, followed by the measurement of TNF-α in the supernatant of negatively selected monocytes. Thereby, the concentration of TNF-α was higher than in the supernatant of positively selected monocytes, irrespective of the GM-CSF activation (Figure 5D). Furthermore, the TNF-α secretion was increased in activated monocytes after negative selection (Figure 5D).

## 4. Discussion

This study investigated the effect of positive and negative magnetic cell sorting on the functionality of isolated human peripheral monocytes after ex vivo culture under non-adherent conditions.

As expected, the positive selection resulted in a higher cell count of monocytes and a higher purity than the negative selection. The higher purity might be related to the increased specificity of the anti-CD14 antibodies. In contrast, with negative selection, CD14^+^ cells remain “untouched” in the supernatant while other cells such as natural killer (NK) cells, T cells, B cells, and granulocytes are captured by antibody-conjugated beads and subsequently removed by magnetic capture. After negative selection, high amounts of small particles (<5 µm) were detected. This seems to be related to the composition of BCs, as they contain higher concentrations of lymphocytes and platelets in contrast to whole blood. High concentrations of platelets were also described after negative selection from isolated peripheral blood mononuclear cells (PBMCs) [18]. After the negative isolation of monocytes from whole human blood, the number of platelets was much lower (Supplementary Figure S1).

Despite the higher purity after positive selection, our analyses revealed a loss of monocyte functionality during ex vivo culture, evidenced by a reduced ability to migrate and adhere as well as a reduced proinflammatory response to LPS. The impaired functionality might be associated with the reduced number of viable cells combined with reduced cell growth. In particular, the cell size shrinking, evidenced by a shift toward the range of dead or dying cells, suggests the start of cell death of the monocytes during the cultivation under non-adherent conditions, which was observed in both naïve and activated monocytes. In addition, the reduced metabolic activity after antibody-based sorting indicates restricted cell growth and differentiation [32,33,34] compared to negatively selected cells. One reason for the preserved functionality after negative magnetic sorting may be the described anti-apoptotic and pro-survival effects by platelets in monocyte cultures due to the secretion of specific chemokines such as C-X-C motif chemokine ligand 12 [35] or platelet factor 4 [36]. However, our additional experiments on monocytes isolated from fresh blood revealed that viability remains high for at least 40 h after isolation (around 90%), even in the absence of high concentrations of platelets (Supplementary Figure S1). Therefore, we assume that the impaired functionality may result from the antibody binding during positive selection, resulting in activation of monocytes. This may have effects on their function and differentiation as previously described for in vitro generated monocyte-derived dendritic cells [20]. The antibodies used for positive selection bind to the CD14 receptor of monocytes, which is an important signaling co-receptor of the Toll-like receptor 4 (TLR4), inducing (among other things) the nuclear factor κB (NF-κB) inflammatory pathway [37]. In our study, the positive selection induced a significant release of the proinflammatory cytokine TNF-α in naïve monocytes, indicating an antibody-based inflammatory activation of TLR4-related pathways. TNF-α was also increased in GM-CSF-activated monocytes, whereby no significant differences were found between positive and negative selection. The concentration of IL-8 in the supernatant of naive monocytes after 16 h of cultivation was unaffected by the isolation method. However, GM-CSF induced the secretion of IL-8 in negatively but not in positively isolated monocytes, indicating attenuated responsiveness to GM-CSF by the preceding antibody binding. The question arises of whether the antibody-induced activation of monocytes impairs the downstream response of monocytes to LPS. Therefore, the monocytes were harvested after ex vivo culture and stimulated with LPS for an additional 24 h. The results show that the negatively selected monocytes secreted significantly more TNF-α than the positively selected monocytes; confirming previously described findings of monocytes cultured under standard (adherent) conditions [38]. In our study, the reduced proinflammatory response may be related to the observed reduced viability and also reflected by the reduced metabolic activity after 16 h of cultivation. Additionally, an LPS tolerance induced by the potential costimulation of TLR4 by CD14 antibodies [39] may explain the diminished TNF-α response. There are indications that the ligation of CD14 antibodies to the cell membrane results in the internalization of TLR4 [19]. Thus, the down-regulation of TLR-4 surface expression may also explain the reduced LPS responsiveness observed in our study. 

## 5. Conclusions

Our functional tests reveal that the isolation by beads-conjugated antibodies is associated with a loss of functionality. In contrast, negative magnetic sorting preserves monocyte-specific functions such as migratory capacity, adherence, or response to inflammatory stimuli. The altered functionality after positive magnetic sorting might be related to inflammatory pre-activation, pro-apoptotic processes, or an interaction of both factors. Depending on the planned applications, possible effects of isolation on monocyte functionality should be carefully considered.

## Data Availability

Not applicable.

## Supplementary Materials

The following supporting information can be downloaded at www.mdpi.com/article/10.3390/biology11111583/s1, Figure S1: Viability after negative magnetic sorting from fresh blood of ten healthy donors.

## References

[1] Jakubzick C.V., Randolph G.J., Henson P.M. (2017). Monocyte differentiation and antigen-presenting functions. Nat. Rev. Immunol..

[2] Coillard A., Segura E. (2019). In vivo differentiation of human monocytes. Front. Immunol..

[3] Herold J., Tillmanns H., Xing Z., Strasser R.H., Braun-Dullaeus R.C. (2006). Isolation and transduction of monocytes: Promising vehicles for therapeutic arteriogenesis. Langenbeck’s Arch. Surg..

[4] De Palma M., Mazzieri R., Politi L.S., Pucci F., Zonari E., Sitia G., Mazzoleni S., Moi D., Venneri M.A., Indraccolo S. (2008). Tumor-targeted interferon-α delivery by Tie2-expressing monocytes inhibits tumor growth and metastasis. Cancer Cell.

[5] Lebson L., Nash K., Kamath S., Herber D., Carty N., Lee D.C., Li Q., Szekeres K., Jinwal U., Koren J. (2010). Trafficking CD11b-positive blood cells deliver therapeutic genes to the brain of amyloid-depositing transgenic mice. J. Neurosci..

[6] Däbritz J., Weinhage T., Varga G., Wirth T., Walscheid K., Brockhausen A., Schwarzmaier D., Bruckner M., Ross M., Bettenworth D. (2015). Reprogramming of monocytes by GM-CSF contributes to regulatory immune functions during intestinal inflammation. J. Immunol..

[7] Weinhage T., Däbritz J., Brockhausen A., Wirth T., Brückner M., Belz M., Foell D., Varga G. (2015). Granulocyte macrophage colony-stimulating factor-activated CD39^+^/CD73^+^ murine monocytes modulate intestinal inflammation via induction of regulatory T cells. Cell. Mol. Gastroenterol. Hepatol..

[8] Green D.S., Nunes A.T., Tosh K.W., David-Ocampo V., Fellowes V.S., Ren J., Jin J., Frodigh S.-E., Pham C., Procter J. (2019). Production of a cellular product consisting of monocytes stimulated with Sylatron^®^ (Peginterferon alfa-2b) and Actimmune^®^ (Interferon gamma-1b) for human use. J. Transl. Med..

[9] Smith C., Økern G., Rehan S., Beagley L., Lee S.K., Aarvak T., Schjetne K.W., Khanna R. (2015). Ex vivo expansion of human T cells for adoptive immunotherapy using the novel Xeno-free CTS immune cell serum replacement. Clin. Transl. Immunol..

[10] Ghaffari S., Torabi-Rahvar M., Aghayan S., Jabbarpour Z., Moradzadeh K., Omidkhoda A., Ahmadbeigi N. (2021). Optimizing interleukin-2 concentration, seeding density and bead-to-cell ratio of T-cell expansion for adoptive immunotherapy. BMC Immunol..

[11] Pakala R., Benedict C.R. (1999). Endothelial cells regulate the proliferation of monocytes in vitro. Atherosclerosis.

[12] Jenkins S.J., Knipper J.A., Zaiss D.M. (2020). Local proliferation of monocytes. J. Leukoc. Biol..

[13] Bennett S., Breit S.N. (1994). Variables in the isolation and culture of human monocytes that are of particular relevance to studies of HIV. J. Leukoc. Biol..

[14] de Almeida M.C., Silva A.C., Barral A., Barral Netto M. (2000). A simple method for human peripheral blood monocyte isolation. Mem. Inst. Oswaldo Cruz.

[15] Fluks A. (1981). Three-step isolation of human blood monocytes using discontinuous density gradients of Percoll. J. Immunol. Methods.

[16] Bøyum A. (1983). Isolation of Human Blood Monocytes with Nycodenz, a New Non-Ionic lodinated Gradient Medium. Scand. J. Immunol..

[17] Klinder A., Markhoff J., Jonitz-Heincke A., Sterna P., Salamon A., Bader R. (2019). Comparison of different cell culture plates for the enrichment of non-adherent human mononuclear cells. Exp. Ther. Med..

[18] Nielsen M.C., Andersen M.N., Møller H.J. (2020). Monocyte isolation techniques significantly impact the phenotype of both isolated monocytes and derived macrophages in vitro. Immunology.

[19] Kim D., Kim J.Y. (2014). Anti-CD14 antibody reduces LPS responsiveness via TLR4 internalization in human monocytes. Mol. Immunol..

[20] Elkord E., Williams P.E., Kynaston H., Rowbottom A.W. (2005). Human monocyte isolation methods influence cytokine production from in vitro generated dendritic cells. Immunology.

[21] Delirezh N., Shojaeefar E., Parvin P., Asadi B. (2013). Comparison the effects of two monocyte isolation methods, plastic adherence and magnetic activated cell sorting methods, on phagocytic activity of generated dendritic cells. Cell J..

[22] Wirthgen E., Hornschuh M., Wrobel I.M., Manteuffel C., Däbritz J. (2021). Mimicking of Blood Flow Results in a Distinct Functional Phenotype in Human Non-Adherent Classical Monocytes. Biology.

[23] Kelley J.L., Rozek M.M., Suenram C.A., Schwartz C.J. (1987). Activation of human blood monocytes by adherence to tissue culture plastic surfaces. Exp. Mol. Pathol..

[24] Tsubota Y., Frey J.M., Raines E.W. (2014). Novel ex vivo culture method for human monocytes uses shear flow to prevent total loss of transendothelial diapedesis function. J. Leukoc. Biol..

[25] Hamilton J.A. (2008). Colony-stimulating factors in inflammation and autoimmunity. Nat. Rev. Immunol..

[26] Hamilton J.A. (2020). GM-CSF in inflammation. J. Exp. Med..

[27] de Waal Malefyt R., Abrams J., Bennett B., Figdor C.G., De Vries J.E. (1991). Interleukin 10 (IL-10) inhibits cytokine synthesis by human monocytes: An autoregulatory role of IL-10 produced by monocytes. J. Exp. Med..

[28] van der Bruggen T., Nijenhuis S., van Raaij E., Verhoef J., Sweder van Asbeck B. (1999). Lipopolysaccharide-induced tumor necrosis factor alpha production by human monocytes involves the raf-1/MEK1-MEK2/ERK1-ERK2 pathway. Infect. Immun..

[29] R Core Team (2021). R: A Language and Environment for Statistical Computing.

[30] Lenth R., Singmann H., Love J., Buerkner P., Herve M. (2018). Emmeans: Estimated Marginal Means, Aka Least-Squares Means.

[31] Kramer C.Y. (1956). Extension of multiple range tests to group means with unequal numbers of replications. Biometrics.

[32] Palmer C.S., Crowe S.M. (2014). How does monocyte metabolism impact inflammation and aging during chronic HIV infection?. AIDS Res. Hum. Retrovir..

[33] Suzuki H., Hisamatsu T., Chiba S., Mori K., Kitazume M.T., Shimamura K., Nakamoto N., Matsuoka K., Ebinuma H., Naganuma M. (2016). Glycolytic pathway affects differentiation of human monocytes to regulatory macrophages. Immunol. Lett..

[34] Groh L., Keating S.T., Joosten L.A., Netea M.G., Riksen N.P. (2018). Monocyte and macrophage immunometabolism in atherosclerosis. Semin. Immunopathol..

[35] Chatterjee M., von Ungern-Sternberg S.N., Seizer P., Schlegel F., Büttcher M., Sindhu N., Müller S., Mack A., Gawaz M. (2015). Platelet-derived CXCL12 regulates monocyte function, survival, differentiation into macrophages and foam cells through differential involvement of CXCR4–CXCR7. Cell Death Dis..

[36] Scheuerer B., Ernst M., Dürrbaum-Landmann I., Fleischer J., Grage-Griebenow E., Brandt E., Flad H.-D., Petersen F. (2000). The CXC-chemokine platelet factor 4 promotes monocyte survival and induces monocyte differentiation into macrophages. Blood J. Am. Soc. Hematol..

[37] Arroyo-Espliguero R., Avanzas P., Jeffery S., Kaski J.C. (2004). CD14 and toll-like receptor 4: A link between infection and acute coronary events?. Heart.

[38] Bhattacharjee J., Das B., Mishra A., Sahay P., Upadhyay P. (2017). Monocytes isolated by positive and negative magnetic sorting techniques show different molecular characteristics and immunophenotypic behaviour. F1000Res.

[39] Mengozzi M., Fantuzzi G., Sironi M., Bianchi M., Fratelli M., Peri G., Bernasconi S., Ghezzi P. (1993). Early down-regulation of TNF production by LPS tolerance in human monocytes: Comparison with IL-1 beta, IL-6, and IL-8. Lymphokine Cytokine Res..

