# Supplementary material for: Negative Magnetic Sorting Preserves the Functionality of Ex Vivo Cultivated Non-Adherent Human Monocytes
Source: Biology (Basel). 2022 Oct 27;11(11):1583. doi: 10.3390/biology11111583 (PMC9687732; doi:10.3390/biology11111583)
Supplement: Supplementary file 1 [file biology-11-01583-s001.zip › biology-1954373-supplementary.pdf]

Supplementary Material

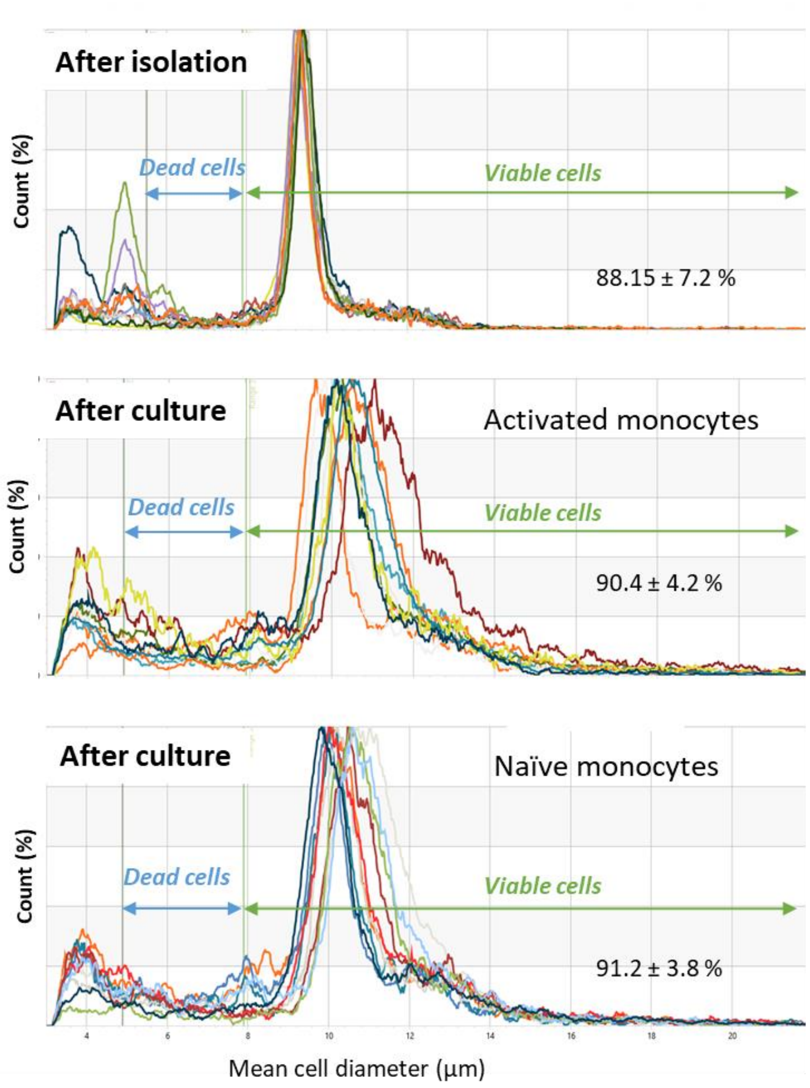

Supplementary Figure S1. Viability after negative magnetic sorting from fresh whole blood of ten healthy donors (approval number: A2021-0229, Rostock University Ethics committee). Isolated cells were measured with CASY counter and calculated as described in detail in Wirthgen et al., 2021. Viability was calculated directly after isolation and after 40 h of culture with GM-CSF (activated) or without stimulation (naïve) as described in detail in (1).

1. Wirthgen E, Hornschuh M, Wrobel IM, Manteuffel C, Däbritz J. Mimicking of Blood Flow Results in a Distinct Functional Phenotype in Human Non-Adherent Classical Monocytes. Biology. 2021;10(8):748.
